# Der Diskurs um § 218 StGB seit der deutschen Wiedervereinigung – Geschichtliche, rechtliche und ethische Aspekte des Schwangerschaftsabbruchs

**DOI:** 10.1007/s00103-024-03992-5

**Published:** 2024-12-11

**Authors:** Florian Michael Dienerowitz

**Affiliations:** Fachgebiet Geschichte, Theorie und Ethik der Medizin, Medizinische Fakultät Mannheim, Universität Heidelberg, Ludolf-Krehl-Straße 13–17, 68167 Mannheim, Deutschland

**Keywords:** Beratungsregelung, Fristenregelung, Reproduktive Rechte, Selbstbestimmung, Lebensrecht, Pregnancy conflict counseling, Maternal-fetal conflict, Reproductive rights, Self-determination, Right to live

## Abstract

Der Schwangerschaftsabbruch ist bis heute Gegenstand harter Diskussionen. Der Beitrag gibt einen Überblick über geschichtliche, ethische und rechtliche Aspekte der Abtreibungsdebatte in Deutschland mit dem Fokus auf die Entwicklungen seit der Wiedervereinigung. Dabei wird die Reform der §§ 218–219 StGB aus dem Jahr 1995 mit der Beratungsregelung im Zentrum beleuchtet. Auch werden Folgen der medizinischen Indikation und juristische Spannungsfelder thematisiert. Vor dem Hintergrund einer sich abzeichnenden Transformation des Schwangerschaftsabbruchs von einem strafrechtlichen Verbot zu einem reproduktiven Recht werden abschließend verschiedene rechtsphilosophische Positionen und praktische Problemstellungen der aktuellen Debatte dargestellt.

## Abstract (en)

Abortion continues to be a highly contentious issue. This article provides an overview of the historical, ethical, and legal aspects of the abortion debate in Germany with a focus on the developments since the reunification. It delves into the background of the 1995 reform of the German abortion law, which centers around mandatory counseling before having an abortion. Furthermore, it outlines the implications of the medical indication for abortion and legal areas of tension. In light of the emerging shift in the status of abortion from a prohibited act under criminal law to a reproductive right, the article presents various legal viewpoints and practical considerations in the ongoing discussion.

## Einleitung

Am 01.10.1995 trat das *Schwangeren- und Familienhilfeänderungsgesetz* in Kraft, das bis heute in weitestgehend unveränderter Form die gesetzlichen Regeln für die jährlich über 100.000 erfassten Schwangerschaftsabbrüche vorgibt. Demnach können Frauen nach Beratung und einer 3‑tägigen Bedenkzeit innerhalb der ersten 12 Schwangerschaftswochen nach Befruchtung einen solchen Abbruch straffrei durchführen lassen (§ 218a Abs. 1 StGB). Außerdem besteht die Möglichkeit, bei medizinischer Indikation (§ 218a Abs. 2 StGB) sowie bei kriminologischer Indikation (§ 218a Abs. 3 StGB) die Schwangerschaft zu beenden.

Aktuell hat die Diskussion um das Abtreibungsstrafrecht, das aufgrund des schwer errungenen Kompromisses zwischen Lebensschutz und Entscheidungsfreiheit der Frau fast ein Vierteljahrhundert lang als nahezu unantastbar galt, neu an Fahrt aufgenommen: In Politik, Wissenschaft und Gesellschaft sprechen sich linksliberale Vertreter meist für eine Entkriminalisierung des Schwangerschaftsabbruchs aus, während konservative Kräfte die aktuellen Regelungen beibehalten oder sogar verschärfen wollen. In Anbetracht dieser möglichen Umbrüche soll im Folgenden ein orientierender Überblick rund um den Diskurs um § 218 StGB seit der deutschen Wiedervereinigung gegeben werden. Dabei werden vor allem Hintergründe und Folgen der Beratungsregelung und der medizinischen Indikation thematisiert, aber auch das juristische Konfliktfeld von Abtreibungen dargestellt. Vor dem Hintergrund einer sich abzeichnenden Transformation des Schwangerschaftsabbruchs von einem strafrechtlichen Verbot zu einem reproduktiven Recht werden abschließend verschiedene rechtsphilosophische Positionen und praktische Problemstellungen der aktuellen Debatte beleuchtet.

## Rechtsgeschichtliche Entwicklungen bis 1990

In der griechisch-römischen Kultur war der Schwangerschaftsabbruch eine weitestgehend akzeptierte Praxis, so befürworteten Platon und Aristoteles Abtreibungen unter anderem als Mittel zur Wahrung idealer Bevölkerungszahlen [1]. Bedingt durch den zunehmenden Einfluss des Christentums verlor der Schwangerschaftsabbruch seine gesellschaftliche Akzeptanz, wobei die von Aristoteles begründete und von bedeutenden Kirchenvätern ebenfalls vertretene Sukzessivbeseelungslehre früh Eingang in das kanonische Rechtsverständnis fand: Während Abtreibungen der nicht vollständig beseelten Leibesfrucht eine schwere Sünde darstellten, galten Abbrüche nach der Beseelung am 40. bzw. 80./90. Tag^1^ als Mord und wurden mit Exkommunikation und der Todesstrafe belegt. Eine (kirchengeschichtlich strittige) Ausnahme davon wurde jedoch bei Gefahr für das Leben der Mutter gewährt.

Das kanonische Recht beeinflusste auch die weltliche Gesetzgebung. So unterscheidet auch die *Peinliche Gerichtsordnung (Constitutio Criminalis Carolina)*^2^ unter Kaiser Karl V. (1500–1558) aus dem Jahr 1532, die als das erste allgemeine deutsche Strafgesetzbuch gilt, bei Abbrüchen zwischen „lebendigem“ und „nicht lebendigem“ Kind, wobei der die beiden Zustände abgrenzende Belebungszeitpunkt nicht genauer definiert wurde. Nicht nur die *Constitutio Criminalis Carolina* hatte bedeutenden Einfluss auf die verschiedenen Partikularstrafgesetzbücher der Frühen Neuzeit, sondern auch naturwissenschaftliche Erkenntnisse zur vorgeburtlichen Entwicklung und nationalstaatliches Gedankengut [2, 3]. So wurden im *Allgemeinen Landrecht für Preußische Staaten* von 1794 die Strafmaße zwar reduziert, jedoch in Abgrenzung zur Sukzessivbeseelungslehre dem Ungeborenen die „allgemeinen Rechte der Menschheit“ ab dem Zeitpunkt der Empfängnis zugesprochen [4]. Andere Gesetze, wie beispielsweise das Bayerische Strafgesetzbuch von 1813, verboten Schwangerschaftsabbrüche hauptsächlich aus bevölkerungspolitischen Gründen zur Stärkung staatlicher Interessen und nicht aufgrund eines etwaigen Lebensrechts des Ungeborenen [3].

Maßgeblich vorgeprägt durch das preußische Strafgesetzbuch von 1851 wurden Abtreibungen im 1871 gegründeten Deutschen Reich ohne Differenzierung des Schwangerschaftszeitpunkts unter § 218 StGB bei den „Straftaten gegen das Leben“ verortet und mit Zuchthausstrafen für die Schwangere und ausführende Dritte belegt [5]. Wenngleich Abbrüche aufgrund wirtschaftlicher Not keine Seltenheit waren, gab es nur wenige Verurteilungen. Zu Beginn des 20. Jahrhunderts wurde zunehmend durch divergierende politische Strömungen und konträre ideologische Überzeugungen die bis heute währende Abtreibungsdebatte angestoßen: Zu nennen sind hierbei die aufkommende Frauenbewegung, die Sozialdemokratie und der Kommunismus, aber auch Sozialdarwinismus und Rassenhygiene sowie der in Anbetracht hoher Menschenverluste im Ersten Weltkrieg vorherrschende Pronatalismus. In der Weimarer Republik gab es bedeutende Protestaktionen und starke Reformbestrebungen des linken Parteienspektrums – die KPD kämpfte sogar nach dem Vorbild der Sowjetunion mit Slogans wie „Dein Körper gehört dir“ für eine Streichung des § 218 StGB [6]. Allerdings wurden 1926 lediglich die Strafmaße reduziert und die in der Praxis ohnehin vielfach angewendete medizinische Indikation durch eine Reichsgerichtsentscheidung 1927 bestätigt [3, 7]. Die Nationalsozialisten machten 1933 die Liberalisierungsansätze mit Blick auf die vermeintlich geschwächte „deutsche Volkskraft“ rückgängig und illegale Schwangerschaftsabbrüche gesunder Kinder wurden durch strenge Kontrollmechanismen vermehrt bekämpft [8, 9]. Die Auslöschung „unerwünschten“ Lebens aufgrund von Rasse oder Behinderung wurde jedoch bereits im Mutterleib angestrebt, sodass 1935 dem sozialdarwinistischen Gedankengut entsprechend eine eugenische Abtreibungsindikation Eingang ins Strafgesetz fand und durch einen Geheimerlass ab 1940 Frauen bei vermuteter Erbkrankheit des Ungeborenen oder aus rassenhygienischen Gründen zur Abtreibung gezwungen werden konnten [3, 10]. Nach dem Zweiten Weltkrieg wurden unter den Siegermächten die nach 1933 erlassenen Strafverschärfungen aufgehoben [11]. Schwangerschaftsabbrüche wurden aufgrund von medizinischer und – in Anbetracht vieler Vergewaltigungen durch Soldaten der Siegermächte – bei kriminologischer Indikation gestattet. In der sowjetischen Ostzone waren die Regelungen noch liberaler, so waren Abtreibungen auch aus eugenischen und sozialen Gründen möglich. 1950 wurden in der 1949 gegründeten DDR die Abbruchmöglichkeiten jedoch aus bevölkerungspolitischen Gründen auf medizinische und eugenische Gründe reduziert, bis man 1972 eine Fristenregelung einführte, bei der die Frau innerhalb der ersten 3 Schwangerschaftsmonate faktisch ein Recht auf Abtreibung hatte [12].

Am 23.05.1949 wurde das Grundgesetz für die Bundesrepublik Deutschland verkündet. Dieses Grundgesetz und seine Auslegung – insbesondere die Artikel 1 Absatz 1 („Die Würde des Menschen ist unantastbar“) und Artikel 2 Absatz 2 („Jeder hat das Recht auf Leben“) – sollten im weiteren Verlauf der Geschichte der Bundesrepublik elementar für die Frage nach der gesetzlichen Handhabung des Schwangerschaftsabbruchs werden. In Bezug auf Schwangerschaftsabbrüche wurde der rechtliche Zustand von 1926 weitgehend wiederhergestellt und somit die Gesetzeslage der Weimarer Republik übernommen. Der Druck hinsichtlich Reformen war in den Nachkriegsjahren durch eine Rückbesinnung auf eine konservative Sexualmoral gering. Erst mit der linksorientierten Studentenbewegung und der sogenannten sexuellen Revolution in den 1960er-Jahren wurden Forderungen nach einer Abschaffung des § 218 StGB laut. Durch verschiedene Protestaktionen wurden Abtreibungen als erweiterte Verhütungsmethode und als ein Recht der Frau eingefordert [13]. Begünstigt durch die Regierungsübernahme der sozialliberalen Koalition aus SPD und FDP im Herbst 1969, die – im Gegensatz zu den zuvor noch in der Großen Koalition von 1966 bis 1969 mitregierenden Unionsparteien – den Schwangerschaftsabbruch weniger kritisch sah, fanden die Forderungen aus Teilen der Bevölkerung Gehör in der Politik. Einem Strafrechtsentwurf einer Gruppe liberaler Strafrechtsprofessoren aus dem Jahr 1970 folgend wurde 1974 eine Fristenregelung mit genereller Straffreiheit in den ersten 3 Schwangerschaftsmonaten verabschiedet [14]. Die Regelung wurde aber 1975 im *1. Fristenregelungsurteil* aufgrund einer Klage der Unionsparteien durch den Zweiten Senat des Bundesverfassungsgerichts gekippt, unter anderem weil auch das Ungeborene das Recht auf Leben besitze und dieses bei einer Fristenregelung in den ersten 3 Monaten nicht gewährleistet sei [15]. Eine Indikationsregelung mit Straffreiheit in Fällen kriminologischer, medizinscher, eugenischer Indikation und sozialer Notlage wurde jedoch höchstrichterlich akzeptiert, was in dieser Form 1976 auch gesetzliche Umsetzung erfuhr [16]. Die Reaktionen auf die Reform fielen unterschiedlich aus: Linksliberale Gruppierungen und Teile der Frauenbewegung zeigten sich enttäuscht, weil das Ziel der völligen Selbstbestimmung der Frau über ihren Körper verfehlt worden sei; große öffentlichkeitswirksame Protestaktionen blieben jedoch aus. Konservative und die katholische Kirche kritisierten die Liberalisierungen hingegen scharf [3].

## Die Gesetzesreformen nach der Wiedervereinigung

Im Rahmen der Wiedervereinigung sah der Einigungsvertrag eine gesamtdeutsche Neuregelung vor [17]. In einem fraktionsübergreifenden Kompromiss wurde 1992 unter der Prämisse „Hilfe statt Strafe“ erneut eine Fristenregelung verabschiedet, bei der neben medizinischer, kriminologischer und eugenischer Indikation Abbrüche bis zur 12. Schwangerschaftswoche nach der Befruchtung nach vorheriger Beratung nicht rechtswidrig sein sollten [18]. Wegen verfassungsrechtlicher Bedenken kam es durch Abgeordnete der CDU/CSU erneut zur Klage vor dem Bundesverfassungsgericht gegen das Gesetz [19].

Tatsächlich widersprach am 28.05.1993 der Zweite Senat des obersten deutschen Gerichts im *2. Fristenregelungsurteil* der Neuregelung in vielen Punkten und bestätigte sein Urteil von 1975, indem er noch detaillierter ausführte, dass Menschenwürde und Lebensrecht nicht erst nach der Geburt, durch eine ausgebildete Personalität oder durch die Annahme der Mutter zustünden. Vielmehr seien sie dem Ungeborenen spätestens ab dem Zeitpunkt der Nidation aufgrund seiner Existenz unabhängig von seinem Entwicklungsstand und ungeachtet religiöser und philosophischer Überzeugungen gegeben [20]. Dementsprechend seien Schwangerschaftsabbrüche außer bei medizinischer oder kriminologischer Indikation stets als rechtswidrig zu klassifizieren [21]. Denkbar sei allerdings, dass präventive Maßnahmen zum Schutz des ungeborenen Lebens besser geeignet sein könnten als Strafen [22]. Das Gericht führte die Bedingungen für eine Neuregelung detailliert aus und erklärte einen Wechsel des Schutzkonzeptes von der vormals bestehenden Indikationsregelung der BRD hin zu einer Beratungsregelung für verfassungskonform. Bei einer solchen Regelung haben Abtreibungen innerhalb einer 12-Wochen-Frist nach der Befruchtung und nach einer staatlich anerkannten Konfliktberatung trotz verfassungsrechtlich gebotener Rechtswidrigkeit keine strafrechtlichen Konsequenzen – die Entscheidung und Letztverantwortung liegt bei der Schwangeren. Sie kann so formell zwar rechtswidrig, aber dennoch rechtlich geordnet einen Schwangerschaftsabbruch durchführen lassen.

Die Reaktionen in Gesellschaft und Politik fielen unterschiedlich aus. Während einige die klaren Aussagen des Gerichts in Bezug auf den Schutz des Ungeborenen lobten, waren anderen genau diese zu streng und nicht zeitgemäß. Schwangere in einer Beratung zum Austragen des Kindes zu ermutigen, sei „unverantwortlich“ und zeuge von einem negativen Frauenbild [23]. Manche unterstellten dem Gericht sogar einen „demagogischen Sprachgebrauch“ [24], unter anderem weil das Urteil von „Tötung menschlichen Lebens“ sprach [25]. Juristen mahnten hingegen an, dass aus dem Urteil Konsequenzen folgen würden, die sich nicht in unsere Rechtsdogmatik integrieren ließen [26]. So wies der konservative Strafrechtler Herbert Tröndle (1919–2017) darauf hin, dass man bei einer derartigen Fristenregelung mit Pflichtberatung zwangsläufig die Tötung einer ungewissen Anzahl ungeborener Kinder in Kauf nehme, um andere zu retten – ein unserem Rechtsverständnis sonst fremdes utilitaristisches Prinzip unter Aufgabe des individuellen Schutzanspruchs jedes einzelnen Menschen [27]. Das Urteil weise zahlreiche Widersprüche auf, z. B. dass die formell rechtswidrige Tat einer Tötungshandlung bei Einkommensschwachen staatlich finanziert wird und der Staat die Infrastruktur für etwas zur Verfügung zu stellen hat, das er eigentlich verbietet [28]. Laut dem liberalen Rechtswissenschaftlicher Reinhard Merkel sei es irrig, „die lediglich verbale, aber rechtlich folgenlose Behauptung eines Grundrechtsstatus des Embryos für eine moralisch höherwertige Position zu halten“ [29].

Der Gesetzesgeber nutzte bei der Umsetzung der Anforderungen des Bundesverfassungsgerichts seine Spielräume zugunsten einer möglichst liberalen Ausgestaltung der Beratungsregelung. Beispielsweise muss nach dem am 29.06.1995 verabschiedeten *Schwangeren- und Familienhilfeänderungsgesetz* der Arzt der Schwangeren lediglich die Gelegenheit bieten, die Gründe für den Abbruchwunsch zu äußern, wohingegen das Gericht dies als verpflichtend voraussetzte [30]. Bayern nahm die Divergenzen zum Anlass, kraft seiner Landeskompetenz ergänzende Regelungen zu schaffen: Unter anderem müssten sich Ärzte die Gründe darlegen lassen und ihre wirtschaftliche Existenz dürfe sich nicht allein auf Schwangerschaftsabbrüche stützen [31]. Gekippt wurde der *Bayerische Sonderweg* vom Ersten Senat des Bundesverfassungsgerichts am 27.10.1998: Der Bundesgesetzgeber habe nicht zu ergänzende Regelungen getroffen. Außerdem verstoße die Reglementierung, nur eine gewisse Zahl an Abbrüchen durchführen zu dürfen, gegen die grundgesetzliche Berufsfreiheit [32]. Der Dissens, der in diesem Urteil zwischen den Auffassungen des Ersten Senats des Bundesverfassungsgerichts und denen des Zweiten Senats in seinem Urteil von 1993 zutage trat, war für viele sinnbildlich für den tiefen Riss, der in der Abtreibungsfrage durch die Gesellschaft geht.

## Die Beratungsregelung, ihre Beratungsstellen und ihr Effekt

Divergenzen in Bezug auf die verschiedenen gesellschaftspolitischen Ansichten zur Abtreibungsfrage spiegeln sich auch bei einer Betrachtung der Beratungsstellen wider. Diese nehmen die zentrale Rolle im Rahmen der Beratungsregelung ein, nach welcher ca. 97 % aller in Deutschland erfassten Schwangerschaftsabbrüche erfolgen. Den staatlich anerkannten Beratungsstellen obliegt die diffizile Aufgabe, einerseits das zentrale Element für den Schutz des ungeborenen Lebens zu sein und andererseits ergebnisoffen zu beraten, um so der schwangeren Frau durch Ausstellung des Beratungsscheins den Weg zur Abtreibung zu ermöglichen [33]. Die zugrunde liegenden Überzeugungen der verschiedenen Träger stellen sich teilweise entgegengesetzt dar, wie die Beispiele von *pro familia* und der katholischen *Caritas* zeigen:

*Pro familia* ist einer der größten Träger von staatlich anerkannten Beratungsstellen und beschäftigt sich ausführlich mit Sexual- und Familienplanungsfragen [34]. Gegründet 1952 und eingebettet in die *International Planned Parenthood Federation* setzt sich der Verein – wie auch die zuletzt genannte Organisation weltweit – für einen uneingeschränkten Zugang zu Schwangerschaftsabbrüchen und somit für eine Streichung der §§ 218–219 StGB ein [35]. Die Pflicht zur Beratung vor einem Abbruch wird als Eingriff in das Selbstbestimmungsrecht der Frau abgelehnt. Ferner unterhält *pro familia* einige Einrichtungen, in denen Beratungen und Abtreibungen als „integrales Dienstleistungsangebot“ unter einem Dach durchgeführt werden [36]. Wenngleich die Anzahl der dort durchgeführten Abtreibungen nicht mit der Größenordnung von Schwangerschaftsabbrüchen in den zahlreichen Abtreibungskliniken der Schwesterorganisation *Planned Parenthood* in den USA zu vergleichen ist, lässt sich eine Divergenz zu den Auffassungen des Bundesverfassungsgerichts erkennen: Es verlangte von den Beratungsstellen nicht nur eine den verfassungsrechtlichen Vorgaben entsprechende Grundeinstellung zum Schutz des ungeborenen Lebens, sondern auch eine organisatorische und wirtschaftliche Trennung von beratenden und abtreibenden Einrichtungen bei möglichen Interessenskonflikten [37]. Dennoch hält *pro familia* das Engagement in der Beratung für unbedenklich: In einer demokratischen Gesellschaft sei es selbstverständlich, Gesetze fachlich und politisch zu kritisieren, um auf Veränderungen hinzuwirken [38].

Ablehnung erfuhr die Beratungsregelung auch durch die katholische Kirche, die anfänglich mit der *Caritas* ebenfalls Teil des Beratungssystems war. Ansatzpunkt für die Kritik war hier aber ein anderer: 1869 hatte sich die Kirche durch einen dogmatischen Erlass von Papst Pius IX. (1792–1878) von der Sukzessivbeseelungslehre entfernt [39]; seither gilt das menschliche Leben vom Augenblick der Empfängnis an als beseelt und ist absolut zu achten und zu schützen [40]. Vor diesem Hintergrund wurde das System, in dem die Beratungsbescheinigung das Zugangsmittel für die straffreie Abtreibung ist, scharf kritisiert. Mit Ausstellung des Beratungsscheins beteilige man sich an der Tötung eines ungeborenen Menschen, weswegen Papst Johannes Paul II. (1920–2005) nach langem innerkirchlichen Diskurs letztlich den Ausstieg aus der staatlich anerkannten Beratung nach §§ 5–10 SchKG zum Jahr 2000 verfügte [41]. Dennoch sahen Teile der Kirche in der Beteiligung am Beratungssystem eine Chance, ungeborenes Leben zu retten, sodass sich katholische Laien – abgekoppelt vom Vatikan – in Form des eigenständigen Vereins *Donum Vitae* als eine staatlich anerkannte Beratung formierten [42].

Nach fast 30-jähriger Anwendung der Beratungsregelung lässt sich Bilanz ziehen: Während in den ersten Jahren der neuen Regelung ein Anstieg der Abbruchzahlen auf über 130.000 pro Jahr zu verzeichnen war, stagnierten sie ab 2013 bei etwa 100.000, seit 2022 gibt es einen erneuten Anstieg. Die relativen Zahlen folgen diesem Kurvenverlauf [26, 43]. Zum Effekt der Beratungsregelung ist hingegen wenig bekannt. So gibt es kaum Informationen zu der Frage, in welchem Maß die Beratungsregelung dazu beiträgt, dass Frauen trotz ihrer Konfliktlage das Kind austragen und wie aktiv die Pflichtberatung angenommen wird. Die Differenz aus erfasster Anzahl von Beratungen und Schwangerschaftsabbrüchen deutet jedoch darauf hin, dass sich knapp ein Drittel der beratenen Frauen gegen einen Schwangerschaftsabbruch entscheidet. Aus Daten von Landesbehörden zu den Beratungen geht zudem hervor, dass Frauen offenbar nur selten von ihrem Recht Gebrauch machen, die Nennung der Konfliktgründe in der Beratung zu verweigern [44, 45].

## Die medizinische Indikation und ihre Folgen

Neben der Beratungsregelung ist die medizinische Indikation ein weiterer Zugangsweg zum Schwangerschaftsabbruch in Deutschland. Zwar macht die medizinische Indikation mit etwa 3 % an der Gesamtheit aller Abbrüche im Vergleich zur Beratungsregelung einen geringen Anteil aus, jedoch birgt sie in vielerlei Hinsicht ihre eigenen rechtlichen und ethischen Herausforderungen. Grundlage der medizinischen Indikation ist § 218a Abs. 2 StGB. Hiernach kann ein Schwangerschaftsabbruch zu jedem Zeitpunkt in der Schwangerschaft erfolgen, sofern ein medizinischer Grund vorliegt. Dieser ist nicht auf lebensbedrohliche Situationen für die Schwangere begrenzt, sondern sehr weitläufig formuliert: Ein Abbruch ist dann nicht rechtswidrig, wenn dieser „unter Berücksichtigung der gegenwärtigen und zukünftigen Lebensverhältnisse der Schwangeren nach ärztlicher Erkenntnis angezeigt ist, um eine Gefahr für das Leben oder die Gefahr einer schwerwiegenden Beeinträchtigung des körperlichen oder seelischen Gesundheitszustandes der Schwangeren abzuwenden“. Wohl auch aufgrund der Aufnahme des Benachteiligungsverbots in Art. 3 Abs. 3 GG am 27.10.1994 wurde im Gegensatz zur Indikationsregelung von 1976 bei der Reform 1995 auf eine eigenständige embryopathische Indikation verzichtet, nach der vormals bei wahrscheinlicher Behinderung des Kindes eine Abtreibung bis zur 22. Woche nach der Empfängnis erfolgen konnte. Während Befürworter der neuen Gesetzeslage den Wegfall der embryopathischen Indikation als wichtige Errungenschaft lobten, mahnten Kritiker an, dass diese zwar in der Gesetzesformulierung, nicht aber in Realität abgeschafft worden sei und sich die Lage von Menschen mit Behinderung dadurch eher verschlimmert habe: So sei die embryopathische Indikation derart verschwommen in der medizinischen Indikation aufgegangen, dass behinderte Kinder allein aufgrund einer unklar definierten Belastung der Schwangeren während der gesamten Schwangerschaft abgetrieben werden könnten [27].

Tatsächlich stellte sich die Problematik von Spätabtreibungen in den Folgejahren als ein wesentliches Defizit der neuen Regelungen dar: Fehlgeschlagene Schwangerschaftsabbrüche, bei denen die Kinder zumindest temporär überlebten, blieben kein Einzelfall. Eines der prominentesten Beispiele ist das sogenannte „Oldenburger Baby“ Tim Guido (1997–2019), der nach der Diagnose einer Trisomie 21 nicht nur den Abbruch in der 25. Schwangerschaftswoche überlebte, sondern auch wider ärztliches Erwarten die Zeit nach der Geburt [46]. Um das Überleben abgetriebener Kinder wie im Fall von Tim Guido zu vermeiden, ging man um das Jahr 2000 dazu über, bei Abtreibungen in späten Schwangerschaftswochen zunächst einen Fetozid durchzuführen: Hierbei wird vor Einleitung der Geburt unter Ultraschallsicht durch Injektion von Kaliumchlorid ins fetale Herz ein Herzstillstand herbeigeführt [47]. Dass dies zu weiteren juristischen und ethischen Herausforderungen führt, zeigt ein Fall aus dem Jahr 2010: Um bei einer Zwillingsschwangerschaft eine tödliche Fehlpunktion zu vermeiden, wurde der selektive Fetozid an dem behinderten Kind erst nach Entbindung seines gesunden Geschwisterchens per Kaiserschnitt an der offenen Gebärmutter durchgeführt. Weil nach Ansicht der Richter die Eröffnung des Uterus die entscheidende strafrechtliche Abgrenzung zu einem aus medizinischen Gründen erlaubten Schwangerschaftsabbruch darstellt, wurden die beiden Ärzte nach § 212 StGB verurteilt [48].

Wenngleich Behindertenverbände und die Ärzteschaft frühzeitig auf ansteigende Zahlen von Spätabtreibungen aufmerksam machten, reagierte die Politik nur zögerlich. Nach jahrelangen Diskussionen trat 2010 eine Änderung des Schwangerschaftskonfliktgesetzes in Kraft: Die ärztliche Beratung bei einer kindlichen Diagnose wurde erweitert, eine ärztliche Hinweispflicht auf das Angebot einer psychosozialen Beratung eingeführt und auch im Falle einer medizinischen Indikation eine Bedenkzeit von 3 Tagen vorgeschrieben, außer bei akuter Lebensgefahr [49, 50]. Da sich die SPD, aber auch Verbände wie *pro familia* gegen Änderungen ausgesprochen hatten, wurde jedoch auf ursprüngliche Forderungen, wie zum Beispiel eine Begrenzung von Abbrüchen bis zur Lebensfähigkeit des Kindes, verzichtet [51, 52]. Die Hoffnung, dass sich durch die kleine Reform Spätabtreibungen reduzieren ließen, bestätigte sich jedoch nicht: Von 2010 bis 2021 stieg die Anzahl der Abbrüche nach der 22. Schwangerschaftswoche von 462 auf 728 bei in diesem Zeitraum insgesamt sinkenden Abbruchzahlen [26].

In jüngerer Zeit versprechen nichtinvasive Pränataltests (NIPT) mittels einer mütterlichen Blutuntersuchung bereits in den ersten Wochen der Schwangerschaft verschiedene Behinderungen des Kindes erkennen zu können. Trotz Kritik an einer hohen Falsch-Positiv-Rate insbesondere bei jüngeren Schwangeren werden die Tests auf Trisomie 13, 18 und 21 seit Juli 2022 von den Krankenkassen übernommen [53, 54]. Diskutiert wird, ob die breite Anwendung der NIPTs langfristig zu einer Verlagerung von Spätabtreibungen auf frühere Schwangerschaftswochen und insgesamt höheren Abtreibungszahlen führt. Wenngleich 2022 und 2023 ein leichter Abfall der Spätabtreibungen bei deutlichem Zuwachs der Gesamtzahl von Schwangerschaftsabbrüchen zu verzeichnen ist, ist ein eindeutiger Zusammenhang schwer zu belegen [43, 55, 56]. Ebenfalls schwer zu überprüfen sind die Befürchtungen, dass aufgrund der NIPTs kaum mehr Kinder mit Trisomie 21 geboren werden könnten [57]: Weder die Zahl der geborenen noch der abgetriebenen Menschen mit Down-Syndrom wird in Deutschland erfasst. Faktisch haben jedoch bereits die herkömmlichen diagnostischen Mittel zu einer iatrogenen vorgeburtlichen Selektion geführt: Nach Schätzungen sind 2012 in Deutschland 90–95 % der ungeborenen Kinder nach pränataler Diagnose eines Down-Syndroms abgetrieben worden [58]. In Dänemark und Island, wo Bluttests in Kombination mit anderen Verfahren bereits seit längerer Zeit als Regelleistungen zum Screening auf chromosomale Anomalien zum Einsatz kommen, ist dieser Prozentsatz noch höher [59, 60].

## Die Ärzteschaft im standesethischen und juristischen Konfliktfeld des Schwangerschaftsabbruchs

Als ausführendes Organ von Abtreibungen kommt der Ärzteschaft eine besondere Rolle zu. Der Schwangerschaftsabbruch stellt eine kontroverse ärztliche Tätigkeit dar, was primär in dem biologischen Sonderstellungsmerkmal der Schwangerschaft begründet liegt: Dem – bis zu einem gewissen Schwangerschaftsstadium – unzertrennlichen Verbund zweier Individuen der Spezies Mensch. Einerseits setzen Ärztinnen und Ärzte ihre Fertigkeiten dafür ein, Schwangerschaften und das ungeborene Leben zu begleiten und zu erhalten, andererseits beenden sie in anderen Fällen die Schwangerschaft aufgrund des Wunschs der Frau oder bei (weitgefasster) medizinischer Indikation. Nimmt ein Arzt eine Abtreibung vor oder verschreibt der Schwangeren die entsprechenden Mittel, so wird er faktisch zum Tötenden oder – im Falle des medikamentösen Abbruchs – zum Beihelfer der Tötungshandlung. Das führt zu einem kaum auflösbaren medizinethischen Konflikt: Während es Teil des Medizinethos ist, zu heilen und nicht zu töten, wird die Ärzteschaft im Falle des Schwangerschaftsabbruchs durch die Ausnahmeregelungen des § 218a StGB als einzige Gruppe in der Gesellschaft mit der Durchführung einer Tötungshandlung betraut. Im Zusammenhang mit der Diskussion um reproduktive Rechte der Frau wird diese Aufgabe zunehmend als Verpflichtung von Ärztinnen und Ärzten angesehen und das gesetzliche Weigerungsrecht [61], an einer Abtreibung mitzuwirken, infrage gestellt [62].

Das medizinethische Spannungsfeld der Ärzteschaft hat seine Konsequenzen auch auf juristischer Ebene. Zu nennen ist hier die sogenannte Kind-als-Schaden-Thematik. Dabei handelt es sich um eine seit Jahren etablierte Rechtsprechung, bei der aufgrund unzureichender Beratung und Diagnostik, die zu einem Abbruch hätten führen können, im Fall einer Behinderung des Kindes Ärztinnen und Ärzten Schadensersatzzahlungen und Unterhaltszahlungen angelastet werden [63]. Während Befürworter dieser Rechtsprechung das Selbstbestimmungsrecht und die Lebensqualität der Mutter als besonders schutzwürdig betonen [64], beklagen Kritiker, dass Ärzte im Fall der Geburt eines Kindes ein juristisch viel höheres Risiko trügen, als wenn ein ungeborenes Kind abgetrieben würde: Ein mutmaßlich behindertes, abgetriebenes Kind könne den Arzt nicht mehr anklagen, die vermeintlich durch die Behinderung ihres Kindes geschädigten Eltern schon, so der drastische Vorwurf [65].

Ein weiteres Beispiel, das Ärzte ins Zentrum der juristischen und politischen Abtreibungsdebatte stellte, war der Diskurs um das sogenannte Werbeverbot. Der ehemalige § 219a StGB untersagte „öffentlich, in einer Versammlung oder durch Verbreiten eines Inhalts“ Dienste und Mittel zur Förderung von Schwangerschaftsabbrüchen anzubieten. Auf die Verurteilung einer Allgemeinmedizinerin im Jahr 2017, die auf der Internetseite ihrer Praxis auf die Durchführung von Schwangerschaftsabbrüchen hinwies, folgte eine Reformdebatte, bei der sich SPD, Grüne und Linke für eine Abschaffung des Paragrafen aussprachen, während sich CDU/CSU und AfD gegen Veränderungen positionierten und die FDP für einen Kompromiss warb. Auch in der Ärzteschaft gab es geteilte Meinungen – zunächst votierte man gegen weitreichende Reformen des § 219a StGB (121. Deutsche Ärztetag 2018), dann für eine Streichung (126. Deutsche Ärztetag 2022). Nachdem die Große Koalition 2019 in einem mühsam erstrittenen Kompromiss Ärztinnen und Ärzten unter Beibehaltung des Werbeverbotes den Hinweis erlaubte, Abbrüche durchzuführen, war das Thema in der Regierung aus SPD, Grünen und FDP wenig strittig: Im Juli 2022 wurde § 219a StGB gestrichen [66]. 2024 folgte zudem ein Verbot von offen artikulierter Kritik an Schwangerschaftsabbrüchen in unmittelbarer Nähe von Abtreibungseinrichtungen und Beratungsstellen (sogenannte Gehsteigbelästigung) mit der Intention, betroffene Frauen zu schützen.

## Aktuelle Reformbestrebungen im Kontext der Grundsatzdebatte über den Status des Ungeborenen

Kritiker werteten die Streichung des Werbeverbotes als einen ersten Schritt mit dem Ziel, den Schwangerschaftsabbruch aus dem Strafgesetzbuch zu entfernen [67]. Tatsächlich verspricht der Koalitionsvertrag von SPD, Grünen und FDP aus dem Jahr 2021, Schwangerschaftsabbrüche zu einer kostenfreien Gesundheitsdienstleistung fortzuentwickeln. Eine unter dieser Zielvorgabe von der Regierung ernannte „Kommission zur reproduktiven Selbstbestimmung“ empfahl im April 2024 Schwangerschaftsabbrüche in der Frühphase der Schwangerschaft uneingeschränkt zu erlauben, dabei sei die extrauterine Lebensfähigkeit des Fötus als Grenze für die Rechtmäßigkeit von Abtreibungen zu betrachten. Dem wollen SPD und Grüne folgen: Abtreibungen sollen außerhalb des Strafgesetzbuches geregelt werden, die Pflichtberatung fallen und die Fristen ausgeweitet werden [68]. Außerdem will man dem Problem von mutmaßlich zu wenig Abtreibungsmöglichkeiten und -ärzten entgegentreten, beispielsweise durch Implementierung von Schwangerschaftsabbrüchen in der ärztlichen Ausbildung. Während die ELSA-Studie aus dem Jahr 2024 unter der Leitung der ehemaligen Pro-familia-Vorsitzenden Daphne Hahn zu dem Ergebnis kommt, dass es tatsächlich deutliche Versorgungslücken gebe [69], zeigen Untersuchungen des Gynäkologen Matthias David, Leitlinienkoordinator der S2k-Leitlinie zum Schwangerschaftsabbruch, andere Ergebnisse: Es sei keine angespannte Versorgungslage zu erkennen, auch nicht in den Jahren der COVID-19-Pandemie [70, 71]. Ein deutlicher Anstieg der Abtreibungszahlen 2022 und 2023 scheint das Vorhandensein von entsprechenden Kapazitäten zu bestätigen [43].

Grundlegende Prämisse für die Aufhebung von Restriktionen des Schwangerschaftsabbruchs ist die reproduktive Selbstbestimmung, deren Anwendbarkeit in Bezug auf Abtreibungen kontrovers diskutiert wird. Konsens ist, dass die Rechte des einen durch die Selbstbestimmung des anderen nicht auf unzumutbare Weise beeinträchtigt werden dürfen [72]. Hat das Ungeborene Rechte, so ist ein Abbruch per Definition auf Grundlage einer selbstbestimmten Entscheidung problematisch, da seine Rechte durch seinen resultierenden Tod auf nicht zumutbare und unumkehrbare Weise berührt werden. Kern der medizinethischen Abtreibungsdebatte ist, ob und ab wann das Ungeborene Rechte besitzt. Theoretisch postuliert das Gesetz zwar, dass „das Ungeborene in jedem Stadium der Schwangerschaft … ein eigenes Recht auf Leben hat“ (§ 219 StGB), faktisch gibt § 218a StGB durch unterschiedliche Fristen aber einen im Verlauf der Schwangerschaft zunehmenden Lebensschutz vor, ein striktes Lebensrecht kommt erst dem geborenen Menschen zu [73]. Das Konzept einer zunehmenden Wertigkeit vorgeburtlichen Lebens findet sich bereits in der oben erwähnten, auf Aristoteles zurückgehenden Beseelungslehre, der zufolge Feten erst nach mehreren Wochen und geschlechtsabhängig mit einer menschlichen Geistseele begabt würden [39].

Im heutigen Diskurs finden sich andere Grenzziehungen, so werden je nach Autor und rechtsphilosophischer Position Merkmale wie Schmerzempfinden, extrauterine Lebensfähigkeit oder Kognition genannt. Nach Reinhard Merkel seien Embryonen lediglich wegen ihres Potenzials, sich zu verletzbaren Wesen zu entwickeln, schützenswert – die Erlebensfähigkeit stelle dabei das entscheidende Merkmal dar [74]. Der Rechtsphilosoph Norbert Hoerster entwickelte zu Beginn der 1990er-Jahre das Konzept eines „Überlebensinteresses“, welches von dem Recht auf Leben zu schützen sei. Da man jedoch nicht genau wisse, wann dieses einsetze und die Freigabe von nachgeburtlichen Kindstötungen zu einer Ausuferung festgelegter Grenzen führen könne, schlägt er als die naturgegebene und für die Rechtspraxis pragmatischste Lösung die Geburt als Grenzlinie für ein Tötungsverbot vor [75]. In internationalen bioethischen Debatten wird jedoch auch die postnatale Kindstötung diskutiert [76]. Auch der australische Ethiker Peter Singer erwägt, dass „unter ganz bestimmten Umständen das volle gesetzlich verankerte Recht auf Leben nicht mit der Geburt in Kraft tritt, sondern erst kurze Zeit, vielleicht etwa einen Monat, nach der Geburt“ [77]. Wichtig für moralisches Handeln sei die Unterscheidung von „Person“ und Mitglied der Spezies Homo sapiens. Ein Fötus sei nicht mehr wert als das Leben eines nichtmenschlichen Lebewesens auf einem ähnlichen Stand der Rationalität, des Selbstbewusstseins und der Empfindungsfähigkeit und mangels dieser Attribute keine „Person“ [77].

Gegner eines abgestuften Lebensschutzes kritisieren hingegen, dass der Staat – sofern er eine neutrale Anschauung vertreten wolle – das Zugehörigkeitskriterium zur Gruppe der Grundrechtsträger vor allem an biologischen Grundlagen zu messen habe und nicht an philosophisch-theologischen Überlegungen [78]. Biologisch betrachtet sei das menschliche Leben als ein fließender Entwicklungsprozess definiert, der von der Befruchtung an auch nach der Geburt über die gesamte Lebensdauer bis zum Tod fortbestehe. Einen klaren Zeitpunkt zu finden, ab welchem dem Ungeborenen das Menschsein und somit Würde und Lebensrecht zugesprochen werden könnten, sei folglich nicht möglich [79]: „Wer Mensch ist, ist es von Anfang an. Das Grundgesetz spricht ausnahmslos jedem Menschen dieselbe Menschenwürde zu. Diese Würde liegt auch für den ungeborenen Menschen schlicht in seiner selbstzweckhaften Existenz“ [80]. So sei beispielsweise auch die extrauterine Lebensfähigkeit des Ungeborenen als Grenzmarkierung problematisch: Sie ist kein eindeutiger Zeitpunkt und verschiebt sich mit medizinischem Fortschritt nach vorne. Hingen die Rechte eines Menschen an seiner Lebensfähigkeit, so wären Ungeborene in späteren Schwangerschaftswochen vor wenigen Jahren weniger schützenswerte Menschen als Ungeborene unserer Tage, weil Föten jetzt sehr viel früher überleben können [45]. Orientiere man sich an einem nicht auszuschließenden Schmerzempfinden, wie es seit 2024 Grundlage für den „vorgeburtlichen“ Schutz von Küken ist [81], so sei es äußerst widersprüchlich, die Grenze für Schwangerschaftsabbrüche ausweiten zu wollen: Entgegen überkommener Annahmen diskutiere man in der Wissenschaft die Reizwahrnehmung inzwischen ab der 12. Schwangerschaftswoche [82, 83], ein scharf abgrenzbarer Zeitpunkt ließe sich aufgrund der Kontinuität der Entwicklung jedoch nicht feststellen.^3^ Ein Ausblenden der biologischen Definition menschlichen Lebens mache Lebensrecht und Menschenwürde letztlich von variablen philosophischen, ideologischen oder religiösen Ansichten abhängig. Diese ließen sich jedoch rasant umdefinieren – mit möglicherweise fatalen Folgen: Die Grenzen könnten auch auf das postnatale Leben übertragen werden und dem Menschen zu jedem beliebigen Zeitpunkt aufgrund seiner „Qualitäten“ das Lebensrecht zu- oder abgesprochen werden, so die Kritik [79].

## Der Schwangerschaftskonflikt in der Abtreibungsdebatte

Neben dem historischen und dennoch weiterhin hochaktuellen Diskurs um den Beginn von Leben(srecht) rücken die Hintergründe von Schwangerschaftsabbrüchen vermehrt in die öffentliche Diskussion. Liberalisierungsbefürworter betonen, dass keine Frau eine Entscheidung für einen Schwangerschaftsabbruch treffen würde, wenn sie das nicht selbst wolle [84]. Die verpflichtende Beratung sowie die 3‑tägige Bedenkzeit zwischen Beratung und Eingriff stünden ihrem Recht auf Zugang zu sicheren und diskriminierungsfreien Schwangerschaftsabbrüchen entgegen [85]. Gegner hingegen postulieren, dass in der Realität der Selbstbestimmung enge Grenzen gesetzt seien. Mit weiteren Liberalisierungen bestünde die Gefahr, dass Schwangere vermehrt von ihren Umständen und ihrem Umfeld beeinflusst einen Abbruch durchführen ließen. Das Strafrecht und die vom Bundesverfassungsgericht auferlegte „Austragungspflicht“ [86] könne neben der verpflichtenden Beratung nicht nur als Schutz für das Kind, sondern auch für die Mutter angesehen werden, indem Dritten die (subtile) Argumentationsgrundlage entzogen werde, ein Schwangerschaftsabbruch sei als Regelleistung der Krankenkassen ein völlig unbedenklicher Eingriff. Kritiker weisen zudem darauf hin, dass bei einer konsequenten Anwendung reproduktiver Rechte nicht nur die Mutter, sondern auch der biologische Vater selbstbestimmt seine soziale Vaterschaft bejahen oder ablehnen können müsste. Dies würde jedoch zwangsläufig die vermeintlich selbstbestimmte Entscheidung der Mutter beeinflussen, da ihr damit die grundlegende emotionale und praktische Unterstützung des Partners entzogen würde [45].

Trotz des Konzepts „Hilfe statt Strafe“, das der Beratungsregelung zugrunde liegt, wurden die Gründe für den Schwangerschaftskonflikt in den letzten Jahrzehnten in Deutschland nur wenig betrachtet. Internationale Literatur betont sozioökonomische Probleme und die Komplexität der Entscheidung, bei der für schwangere Frauen die eigenen Bedürfnisse, ein Verantwortungsgefühl gegenüber nahestehenden Personen sowie der Beitrag des genetischen Vaters eine Rolle spielten [87, 88]. Aktuelle auf Deutschland bezogene Veröffentlichungen bestätigen, dass Schwangere einer Vielzahl von äußeren Einflüssen ausgesetzt sind, die einer freien Entscheidung für das Kind entgegenstehen können. Genannt werden Partnerschaftsprobleme, biografische Zwänge, Überforderung und materielle Sorgen [26, 44, 45]. Auch deuten einige aktuelle Untersuchungen und Statistiken darauf hin, dass die direkte und – häufiger noch – die indirekte ablehnende Haltung Dritter, insbesondere des Kindesvaters, für einen nicht unerheblichen Teil von Schwangeren bedeutenden Einfluss hat [45, 89]. Dies wiederum wurde nicht nur von Beobachtern bereits in den 1990er-Jahren als eine scheinbar neutrale Haltung beschrieben, die mit der unausgesprochenen Erwartung verknüpft sei, die Partnerin möge sich im Sinne der Interessen des Mannes für einen Abbruch entscheiden [90], sondern auch vom Bundesverfassungsgericht im Jahr 1993 kritisch beleuchtet: Schwangerschaftskonflikte, die schließlich zum Schwangerschaftsabbruch führen, hätten ihre Ursache zu einem erheblichen Teil in gestörten Partnerschaftsbeziehungen, in der Ablehnung des Kindes durch den Vater oder die Eltern der Frau sowie in einem Druck, der von diesen ausgeübt werde [91]. Personen des familiären und des weiteren sozialen Umfelds würden Schwangere häufig – und nicht selten in strafwürdiger Weise – gegen das Kind beeinflussen [92].

## Fazit und Ausblick

Nachdem das durch das Christentum vorgeprägte und in der Zeit der Aufklärung strafrechtlich verankerte Verbot der Tötung ungeborenen Lebens im Konzept „Hilfe statt Strafe“ in den 1990er-Jahren bereits deutlich zugunsten einer autonomen Entscheidung der Frau verschoben wurde, ist heutzutage ein Wandel zu einem „Recht auf Schwangerschaftsabbruch“ zu beobachten [26]. Traditionell fordern dies linksorientierte Parteien, zunehmend aber auch supranationale Organisationen wie die Vereinten Nationen und das Europäische Parlament. Letzteres verlangte im Juli 2022 und im April 2024 die Aufnahme eines solchen Rechts in die Charta der Grundrechte der Europäischen Union. In Frankreich wurde im März 2024 die „Freiheit zur Abtreibung“ bereits in der Verfassung verankert [93].

Derartiges ist in Deutschland jedoch nicht mit der bisherigen Position des Bundesverfassungsgerichts vereinbar: Dem Ungeborenen sei unabhängig von seinem Entwicklungsstand Menschenwürde und Lebensrecht gegeben: „Wo menschliches Leben existiert, kommt ihm Menschenwürde zu“ [94]. Dies steht einem uneingeschränkten Verfügen der Frau über ihre Leibesfrucht entgegen [95]. Die mit der Beratungsregelung einhergehende Liberalisierung legitimierte das Gericht in seinem Urteil vom 28.05.1993 nur, wenn dadurch ein besserer Schutz des ungeborenen Lebens gewährleistet werde – ansonsten seien gesetzliche Nachbesserungen zu veranlassen [96]. Ein verbesserter Lebensschutz lässt sich nach 30-jähriger Beobachtung der Abbruchzahlen jedoch nicht ablesen: Während in den Jahren 1977 bis 1992 auf dem Gebiet der BRD mit ihrer etwas strengeren Indikationsregel 11,2 % aller Schwangerschaften abgebrochen wurden und in der ehemaligen DDR mit ihrem liberalen „Recht auf Abtreibung“ 29 % (1972–1992), waren es unter dem nach der Wiedervereinigung etablierten Kompromiss zwischen Lebensschutz und Selbstbestimmung von 1996 bis 2023 etwa 13,5 % der Schwangerschaften [26, 45].

Neben dem rechtsphilosophischen Diskurs um reproduktive Selbstbestimmung und den Status des Ungeborenen wird vermehrt diskutiert, ob und inwieweit Liberalisierungen Schwangeren in ihren durch Umstände und Umfeld geprägten Lebensrealitäten konkret weiterhelfen würden. Ein wesentliches Defizit des gesellschaftspolitischen Diskurses ist eine mangelhafte Auseinandersetzung mit den Gründen des Schwangerschaftskonflikts. Nur wenig wurden in den vergangenen Jahrzehnten Konfliktursachen untersucht und daraus effektive (langfristige) Hilfen erarbeitet, die eine Perspektive mit Kind eröffnen könnten [97]. Der ehemalige Bundespräsident Horst Köhler drückte dies folgendermaßen aus: „Niemand weiß, wie viele Kinder allein deshalb am Leben gehindert werden, weil ihre Eltern sich … alleingelassen fühlen. … Tun wir genug dafür, dass junge Menschen frohen Herzens ‚ja‘ sagen können – zu erwünschten genauso wie zu unerwarteten Kindern?“ [98].

## Footnotes

1 40 Tage beziehen sich auf die männliche Leibesfrucht. Für die weibliche Leibesfrucht wird – je nach Quelle – der 80. oder 90. Tag als Zeitpunkt der Beseelung genannt.

2 „Peinlich“ bezieht sich auf lat. „poena“ für „Strafe“.

3 Die wissenschaftliche Debatte um die Reizwahrnehmung am Anfang des Lebens ist in stetigem Wandel. Lange wurde argumentiert, dass Schmerzempfinden frühestens ab der 24. Schwangerschaftswoche möglich sei oder sich sogar erst nach der Geburt entwickele. Während Neugeborene noch in den 1980er-Jahren ohne Analgesie operiert wurden, wird heute in der Pränatalchirurgie eine fetale Schmerztherapie empfohlen.
